# Burden of illness of rheumatoid arthritis in Latin America: a regional perspective

**DOI:** 10.1007/s10067-015-3012-0

**Published:** 2015-07-30

**Authors:** L. A. Ramírez, C. Rodríguez, M. H. Cardiel

**Affiliations:** Sección de Reumatología, Universidad de Antioquia, Medellín, Colombia; Fundación Memuevo, Santiago, Chile; Centro de Investigación Clínica de Morelia, Virrey de Mendoza 1998–Int 522. Col, Félix Ireta, CP 58070 Morelia, Michoacán Mexico

**Keywords:** Costs, Epidemiology, Patient experience, Priority health problems, Rheumatoid arthritis

## Abstract

Rheumatoid arthritis (RA) is one of the most important rheumatic diseases. Its prevalence varies among ethnic groups. Genetic and environmental factors influence its incidence and prevalence. This chronic disease will increase its frequency in the future due to population aging. The personal impact of this disease on many relevant areas of an individual requires special efforts to prevent and treat it properly. Adequate advice on several recently described risk factors such as tobacco and alcohol exposure, infections, obesity, and physical exercise should be part of every medical consultation. This knowledge should be incorporated to improve health care prevention programs. Patients and clinicians must work together through better communication skills to finally improve outcomes. Including RA in priority health care lists will need special effort from rheumatology societies and better communication with policy makers.

Rheumatoid arthritis (RA) is a chronic autoimmune disease of unknown origin. Multiple factors have been proposed to interact to produce a systemic disease that is characterized by joint pain and swelling and a myriad of multiple manifestations outside the joints. If not properly treated, this joint swelling will produce damage and progressive disability. Life expectancy is also diminished [1].

RA prevalence is around 1 % in the world but this number changes in different ethnic populations. In Mexico, one study using COPCORD methodology in 19,213 individuals, in five different regions, found a global prevalence of 1.6 % with a different prevalence rate ranging from 0.7 % (95 % confidence interval (CI): 0.5–1.0) in Nuevo León to 2.8 % (95 % CI: 2.3–3.3) in Yucatán [2]. In Luján, Argentina, a city of 70,000 inhabitants, a prevalence rate of 0.94 % (95 % CI: 0.86–1.02 %) was found.

[3]. An interesting study recently done in 1656 Toba individuals living in Rosario, Argentina, found a prevalence of 2.4 (95 % CI: 1.7–3.2) [4]. These findings of increased prevalence in Yucatán, México, rich in original Mayan population and in Toba population in Argentina have raised increased interest in genetic, anthropologic, and environmental factors in the development of RA in original populations in our region. Multiple studies are currently undergoing in several Latin American countries including Argentina, Mexico, and Venezuela in an initiative called GLADERPO (Grupo LAtinoamericano De Enfermedades Reumáticas en Poblaciones Originales).

## Incidence rates of RA are scarce in Latin America

A recently published study in Ontario in 97,499 Ontarians found a cumulative prevalence of 0.9 %. Age- and sex-standardized RA prevalence increased steadily over time from 473 (95 % CI: 469–478) per 100,000 population (0.49 %) in 1996 to 784 (95 % CI: 779–789) per 100,000 population (0.9 %) in 2010. Age- and sex-standardized incidence per 100,000 population ranged from 62 (95 % CI: 60–63) in 1996 to 54 (95 % CI: 52–55) in 2010 [5].

## RA is the prototype of chronic diseases

It is very important to be aware of multiple risk factors that have been described and intervene in those that are modifiable in order to improve patient outcomes. A brief description of the most commonly cited and recently described is presented.

## Genetics

It is well recognized that rheumatoid arthritis has a clear association with some genetic markers [6]. The shared epitope is one of the most studied but there are other genes that have been identified such as the PTPN22 [7]. Some protective genes have been described in Chinese population from Taiwan [8]. Some new genes have been detected in Latin American populations [9]. Although there have been important research studies in this area, no direct clear benefit has been offered to our patients with this knowledge yet. It is estimated that genes do not explain the great majority of RA cases.

## Environmental factors

Intensive and active research has been given to multiple environmental factors that have been identified as associated with the development of rheumatoid arthritis. The most studied have been hormonal, tobacco, alcohol, infections, toxic and pollutant agents, obesity, and physical exercise. Updated information is presented.

## Tobacco

It has been established that tobacco is a risk factor for RA development and particularly linked with a seropositive disease [10]. It has been described that this association is clearly related with genetic markers. Clinicians should be aware of smoking habits in their patients, and cessation programs should be implemented. It has been suggested that RA patients who continue smoking have less therapeutic clinical response [11].

## Alcohol

Mild to moderate alcohol consumption has been associated with a protective effect on RA development when compared to no or high alcohol consumption [12]. Some possible explanations have been offered, and immune modulation and anti-inflammatory actions are the most plausible theories. Although it is quite difficult to advise alcohol consumption, clinicians could identify the potential benefit of a low to moderate intake that seems to be particularly beneficial in women in a recent meta-analysis [13].

## Infections

An increased interest in infections has gained relevance in the last decade as a risk factor for RA development. There is supporting evidence that *Porphyromonas gingivalis* is associated with seropositive disease due to the citrullination capabilities of this bacterium [14]. Some studies have also shown that this association is related with disease activity [15]. Increased interest in gut microbiome has gained intense research attention in the last years in some diseases such as RA, psoriasis, obesity, and metabolic syndrome. These findings are quite promising since they offer clear practical possibilities for therapeutic interventions.

## Toxic and pollutants

Some studies have identified increased RA in some areas or human activities, where exposure to pollutants has been described. This has been detected in urban areas [16], exposure to insecticides [17], and also related to a low socioeconomic status [18]. The explanation of this interaction has been proposed to be through epigenetic changes in immune response genes. This piece of knowledge will be very important to design possible reversibility of these genetic modifications in the future. A clinician should be aware of this and give proper advice to patients and their families.

## Obesity

This recently described risk factor [19] is very important in Latin America when we are facing increasing obesity problems even starting in childhood. It has been postulated that obese subjects have an increased risk for RA development, and obese subjects have a decreased chance to reach remission or a low disease activity state [20]. Some data are also available describing that obese subjects have a lower rate of joint damage progression that has raised some possible metabolic explanations [21]. This area of intervention is one of the most important to be emphasized in a clinical setting.

## Physical activity

A recently published study described that patients who have been physically active during the past 5 years previous to RA development had a milder disease compared to those subjects who were sedentary [22] in terms of disease activity, pain, and function. This information is particularly useful to be taken into account in an appropriate medical advice and public health recommendations.

## Social and economic impact of rheumatoid arthritis

The increase in life expectancy has raised the frequency of chronic musculoskeletal diseases, such as rheumatoid arthritis (RA), and elevated treatment costs, generating global concern for the last decade, which was declared as the “decade of bone and joint” by the World Health Organization. A statement given by UN Secretary General Kofi Annan, declaring the Bone and Joint Decade 2000–2010, 30 November 1999: “Musculoskeletal disorders are the most common causes of severe long term pain and physical disability, affecting many millions of people across the globe. They have an enormous impact on the individual, society, and health care social systems. There are effective ways to prevent or treat these disabling conditions. But we must act on them now [23].”

RA is a chronic autoimmune disease that increases morbidity, mortality, and produces high economic costs for the patient, the family, and society, besides altering the emotional balance, thus altering the quality of life [24].

Here are some necessary definitions of the costs to clearly refer later:

### Direct costs

They are determined by the derivative of direct patient care, like: medication, hospitalization, diagnostic or therapeutic interventions, professional fees, transportation, mechanical aids for the patient, physiotherapy, as well as nursing value.

### Indirect costs

Are those that come from paying disabilities.

### Intangible costs

They are difficult to estimate, since they result from the reduction in earning capacity or decrease in life expectancy [25, 26].

Without delving exhaustively, costs must be differentiated before and after biologic agents, since these increased between 3–6 times and have moved hospitalization expenses as those responsible for the high cost, as indicated by studies in the USA and Europe [26, 27]. Michaud et al., in their cohort, found a cost of US$6164/year when synthetic disease-modifying antirheumatic drugs (DMARDs) are used, compared to US$19,016/year with biologics [26]. A similar situation was observed in a French study [27]. However, with the introduction of the combined therapy and biologics, we have seen positive results, not only in the clinical aspect but also in the indirect costs that are reflected in decreased hospital days, decreased sick leave, and therefore, decrease of absenteeism, with increased employment of patients, both men and women [28, 29]. Another positive topic, as reported by Huscher et al., is that there is an absence of a steady increase in drug costs, since it is estimated that, after 7 years, a plateau in the curve of costs is reached by biologics [30]. But some doubts remain regarding the possible success, as the sense of well-being and the intensity of pain does not improve over time through a decade [28].

Another relevant aspect is that there are factors of the same disease that increase costs; for example, if the disease has a duration of more or less than 6 months, significant differences are established, with greater cost and with more disease duration, and the same phenomenon occurs with the number of comorbidities [25]. With respect to HAQ, there is an exponential relationship, as a higher score implies higher costs, as much as one point increase represents a cost of US$8084/year [26, 31].

An indirect cost related to AR is the work stoppage. In a study of 133 patients with 3 years of follow-up since the onset of the disease, 78 (59 %) continued working, 23 (17 %) were retired due to RA, 12 (9 %) lost their jobs but did not receive pension, and 20 (15 %) were out of the workforce for reasons other than RA causes [32].

In Latin America, there are few studies on costs in RA, but they generally agree with the above (Table 1). A study in Mexico by Ariza et al. indicates the lowest cost in the region in relation to the exclusive use of synthetic DMARDs [33]; along with the study of Buenogens et al. [34], they are based on real-life patients. A Colombian work, conducted by Mora et al., is a simulation study and represents an important contribution that provides a categorization based on disease activity, demonstrating that high disease activity translates into increased direct cost, fundamental basis in drug costs, becoming similar to those of other first world countries [35].

**Table 1:** Comparison of costs in patients with rheumatoid arthritis in Latin America

Country/year	Total direct costs US$	Drug costs (%)	Medical costs (%)	Other costs (%)	Synthetic DMARDs	Biologic agents
Mexico 2005 (12)	1735	25.76	51.33	22.9	Yes	Do not
Brazil 2008 (13)	14,185	90.8	6.8	2.4	Yes	Yes
Colombia 2009 (14)	1,689–23,441^a^	86	14	–	Yes	Yes

^a^Depending on the rate of activity of the AR

## Rheumatologist perspective

Undoubtedly, in recent decades, there has been a qualitative change in the confrontation of RA, because, in the first place, it was recognized as a severe disease that turned the existing paradigm even in the second half of the last century; this led to design strategies to achieve earlier diagnosis, such as the development of early arthritis clinics that have allowed us not only to distinguish RA from other early inflammatory arthritis but also to recognize patients with more aggressive disease, and, in conjunction with the above statement, to set treatments according to the aggressiveness, such as combination therapy with methotrexate as the cornerstone of treatment. Finally, the appearance of anti-TNF agents and other biologics, which are similar to combination therapy, have been a step forward in searching for a better disease control and, certainly, a better prognosis. We consider that the establishment of new classification criteria for RA will result in strengthening the possibility of an early diagnosis of a serious disease.

Taking this into consideration has led a new positioning of the rheumatologist as the leader of an interdisciplinary team with greater strength, security, and optimism in his results. We all hope that, in the nearest future, the cost of biologic agents will decrease, and with the decrease of indirect and intangible costs, these drugs will be more accessible for the general population.

The above expectations are not free of any danger, since the rheumatologist often feels pressure, not only by health-care providers but also by the patients themselves, who, in their anxiety about their illness and knowledgeable about the existence of biologic therapy, demand for its early use, even without previous evaluation of synthetic DMARDs. We think that their behavior is influenced by the pharmaceutical industry.

Finally, we think that if we compare our current practice with the past, the current scenario will contain much less frustration and hopelessness than the one lived by rheumatologists 40 years ago, and, for patients, the future is safer, even in countries like those in Latin America.

## Illness or disease? A life with rheumatoid arthritis: the words from a patient

A large part of the cultural discord that exists between patients and clinicians lies in the inherent difference between the patients’ subjective experience of “illness” and the clinician’s objective approach to “disease” [36].

We cannot start talking about the experience of living with rheumatoid arthritis without taking some time to look at this definition, written several years ago, but still so current. And this is the case because it is not only a body that gets affected, and it is not just a disease. Living with rheumatoid arthritis means to experience deep transformations in different areas in life: love, family, social life, job, to mention just a few; to live the subjective experience of illness.

One of the narrations shared by patients with rheumatoid arthritis concerns with some questions that arise after the diagnosis: *why*, *why me*, *what did I do wrong*? The attempt to associate a traumatic event and the appearance of the first symptoms is also recurrent, which somehow—true or not—enables you to make sense of the transformation of the SELF many of us go through. Already in the year 1981, Baron stated: “people do not come in for diagnosis and treatment; they come to be made well, made whole, to recover the sense of health, of being well, fully alive, in-the-world [37].”

*Readapting* life is probably a term as valued by patients with rheumatoid arthritis as *remission*, for being well, fully alive implies a learning process, well summarized by this young Chilean patient: “rheumatoid arthritis has given me the gift of *awareness* to accept I am a vulnerable woman, who feels and makes mistakes; *courage* to face my fears; *passion* to live each day as if it was the last, understand life is today and appreciate everything that shows up in my space; *gratitude* to realize I have a wonderful family that takes care of me, and gives me containment and support; and *faith*, because I am certain that arthritis is just the symptom coming to show me the unlearned lesson.”

Achieving a good re-adaptation or learning process depends on at least five factors that we have been able to identify working with these patients:

### Access to treatment

Latin America undergoes a very dissimilar reality with regards to access to therapies. Nevertheless, there is a common feeling concerning the financial decay generated by the difficulty to keep a steady job due to the symptomatology of the disease. An Argentinean young woman tells it with a touch of humor that does not hide how dramatic this situation is: “I don’t know what hurts more, my joints or the fact that what I make is not enough to cover all my expenses. Since I got sick, my financial situation worsened.” Treatment does not only imply medicine; treatment also means to have an appropriate job, where you are still an active member of society, physical therapy, and body re-education, among others.

### Social integration

Counting on family, friends, or coworkers who understand what you go through is comforting. Part of the process each one goes through is that of accepting the new limitations. If they have to be constantly hidden, this acceptance takes more than needed, thus delaying the re-learning process. But the doubt that constantly arises is how to make the other a part of an invisible pain that very often appears and disappears with no explanation? The experience of chronic pain affects the state of mind, and therefore, social relationships.

### Being recognized as “experts”

If we added up the hours we spend with the clinical treating team, they would not add up to more than a couple of days in a year. This condition is with us on a daily basis. Therefore, certainly the ones who know better than anyone else how it manifests, how it affects, or possible ups and downs are the patients themselves. The fear to express something experienced as reality very often affects the adherence to any treatment and, above all, the self-management process that every person living with a chronic condition requires. As defined by Angela Coulter, it is necessary to learn about sharing expertise [37] and summarized in Table 2.

**Table 2:** Patient and clinician share expertise

Patient	Clinician
Expertise of illness	Diagnosis
Social circumstances	Disease etiology
Attitude to risk	Prognosis
Values	Treatment options
Preferences	Outcome probabilities

Understanding and respecting everybody’s scope of action enables the movement towards a healthier and stronger physician-patient relationship. Expert patients are “people who have the confidence, skills, information, and knowledge to play a central role in the management of life with chronic diseases and to minimize the impact of disease on their lives” [38].

### First-hand quality information

Making decisions dealing with one’s own health and life in general is a process whose frequency and motivation very often depend on how rooted this practice is within one’s own culture. When it comes to approaching chronic diseases, this is a central process of self-management. Making decisions requires having the best information available. Most of the time, the patient expects that information to be given by someone in the group where they put their trust: a member of their clinical team. Patients’ and clinicians’ communication skills are critical to pushing forward with this process, so as to select everything that is important, but at the same time, present it in a way that the other can understand.

### Peer support and social networking

The great need to rely on people who understands the process that you are going through has led in recent years to a sharp rise in the amount of social networks available for patients with rheumatoid arthritis. In these virtual groups, not only the support and understanding very often required are found but also experiences regarding the disease are shared, which enable better management. Nevertheless, without a clear understanding of what role each of them plays in the process, situations can come up in which among patients there are recommendations on what drugs or dosage to use. Once again, a good partnership between physicians and patients can build a better virtual space.

Living a life with rheumatoid arthritis is a life in which you have to take charge of an illness and a disease, and to do so, a strong partnership between clinicians and patients is undoubtedly the best strategy.

An integrated approach to rheumatoid arthritis requires not only substantial improvements in public policies in each of our countries but also progress in the clinician-patient relationship towards informed, shared decision-making and patient-centered care. We know it is a long way, but we want to be a part of it. Let us help!^1^

## Rheumatoid arthritis and priority health problems

Rheumatic diseases represent a complex group of health problems that produce diverse degrees of pain, suffering, disability, and poor quality of life. If not properly treated a decreased survival is also detected [1].

Rheumatologists have been struggling for many years in different health care environments to improve patient care. Latin America has important challenges in terms of epidemiologic transition. Our region still has multiple health-care needs that are common to developing countries such as maternal and health-care problems, infections, addictions, violence and at the same time they need to provide adequate care to chronic diseases commonly seen in developed countries such as obesity, diabetes, hypertension, cancer, and rheumatic diseases. A recently published survey on global disability found that RA was ranked as the 42nd highest contributor to global disability, just below malaria and just above iodine deficiency [38].

Health-care budgets need to establish priorities that take into account several approaches [39]. Exclusion of rheumatic diseases is a common finding in these exercises since burden of illness is frequently taken into account and the low prevalence of RA does not compete with other large health-care problems such as hypertension, type 2 diabetes mellitus, malnutrition, and maternal health. Other criteria that include preventive interventions and cost-effective interventions are not easily justified to reach a priority.

Political issues dominate some decisions, and rheumatology societies have not delivered yet solid local information to be used by policy makers.

Smart strategies should be proposed to gather more and better knowledge, incorporate the concept of quality of life in a multi dimensional concept decision process. Rheumatology societies should be part of teams of decision makers and plan organized strategies that need to be consistent with the final ideal of better health from a societal perspective.

Rheumatologists need to be aware that adequate clinical and therapeutic decisions should always take into account the rational use of resources in our patients in the context of societies looking for equity.

Regional decisions should try to produce better outcomes using optimized strategies in our patients. Rheumatologists have a great potential to change this perspective that leaves out rheumatoid arthritis and other rheumatic diseases from priority health list in the region, by performing coherent activities to positively change health care of rheumatic patients in Latin America.

## Footnotes

1 Let us help! is inspired by David deBronkart’s quote “Please, let patients help improve healthcare. Let patients help steer our decisions, strategic and practical. Let patients help define what value in medicine is.”
